# Association of serum uric acid-to-high-density lipoprotein cholesterol ratio with non-alcoholic fatty liver disease in American adults: a population-based analysis

**DOI:** 10.3389/fmed.2023.1164096

**Published:** 2023-05-15

**Authors:** Yilian Xie, Kai Huang, Xiangyu Zhang, Zhouxiao Wu, Yiyi Wu, Jinguo Chu, Weiliang Kong, Guoqing Qian

**Affiliations:** ^1^Department of Infectious Diseases, Ningbo First Hospital, Ningbo, Zhejiang, China; ^2^Department of Hepatology, Ningbo First Hospital, Ningbo, Zhejiang, China; ^3^Department of General Medicine, Ningbo First Hospital, Ningbo, Zhejiang, China; ^4^Department of Nephrology, Ningbo First Hospital, Ningbo, Zhejiang, China; ^5^Department of Respiratory and Critical Care Medicine, Ningbo First Hospital, Ningbo, Zhejiang, China

**Keywords:** UHR, NAFLD, NHANES, steatosis, vibration controlled transient elastography

## Abstract

**Objective:**

Non-invasive disease indicators are currently limited and need further research due to the increased non-alcoholic fatty liver disease (NAFLD) prevalence worldwide. The serum uric acid-to-high-density lipoprotein cholesterol ratio (UHR) has been recognized as a novel inflammatory and metabolic marker. Herein, we explored the correlation between UHR and the risk of NAFLD in-depth.

**Methods:**

A total of 3,766 participants were included in our survey, and the National Health and Nutrition Examination Survey (NHANES) 2017–2018 cycle provided the cross-sectional study population. Weighted multivariable logistic regression and multivariate linear regression analyses were performed to assess the association between the UHR and the odds of NAFLD and liver steatosis and fibrosis severity, respectively. Moreover, we explored the non-linear relationship between the UHR and NAFLD by the generalized additive model.

**Results:**

NAFLD probabilities were statistically demonstrated to be positively correlated with the UHR (OR = 1.331 per SD increase, 95% CI: 1.100, 1.611). The positive connection of the UHR with NAFLD risk persisted significantly in female subjects but not in male subjects in subgroup analyses stratified by gender. The non-linear relationship analysis demonstrated that a UHR between ~20 and 30% suggested a saturation effect of NAFLD risk. Furthermore, a dramatically positive correlation was found between the UHR and hepatic steatosis severity but not fibrosis. Finally, the receiver operating characteristic analysis suggested that UHR had a better predictive value for NAFLD than either serum uric acid (sUA) or high-density lipoprotein cholesterol (HDL) alone [UHR (area under curve): 0.6910; 95% CI: 0.6737–0.7083; *P* < 0.0001].

**Conclusion:**

Our investigation revealed that the elevated UHR level was independently related to an increased NAFLD risk and the severity of liver steatosis in American individuals. The correlation differed according to sex. This non-invasive indicator may enhance the capacity to predict the onset of NAFLD and may uncover alternative therapeutic interventional targets.

## Introduction

The prevalence and incidence of non-alcoholic fatty liver disease (NAFLD) are significantly increasing globally (1–4). At present, the prevalence of NAFLD is from 13% [Africa (1) to 42% southeast Asia (4)] worldwide, of which the prevalence in the United States (US) is 35.3% (5), and is continuing to increase (4). By virtue of its high prevalence, NAFLD is progressively being documented as a major reason of liver cirrhosis, fibrosis, hepatocellular carcinoma (HCC), or even liver transplantation with an enormous socioeconomic burden to society (4, 6), and a rapidly growing cause of liver-related mortality worldwide (3). Therefore, it is important to find an efficient, rapid, affordable biomarker to identify and stage fatty liver early (7).

Non-alcoholic fatty liver disease has been identified as having a strong, bidirectional relationship with dyslipidemia, obesity, hypertension, and type 2 diabetes mellitus (T2DM), as well as representing a hepatic manifestation of the metabolic syndrome (MetS) (2). Similarly, the purine metabolism final product by the liver, serum uric acid (sUA), has been associated with the risk of MetS (8). A reduction in HDL may influence the body's oxidative condition since high-density lipoprotein cholesterol (HDL-C) is a plasma lipoprotein with excellent anti-inflammatory and antioxidant roles (9). Both elevated sUA and reduced HDL-C levels are linked to a higher risk of developing NAFLD, and HDL-C and UA have been suggested to play opposing roles on MetS (10–14). More recently, it was discovered that the sUA-to-HDL-C ratio (UHR) was a novel inflammatory and metabolic marker that was associated with increased metabolic syndrome risk (15–17) and was more predictive of the onset of NAFLD than UA or HDL-C alone (18). There have been few studies linking UHR to the risk of developing NAFLD, and less is recognized about the relationship between UHR and the degree of liver steatosis and fibrosis in American populations (19, 20). Here, we conducted a large cross-sectional study to investigate the relationship between UHR and NAFLD risk in the adult American population using data from the National Health and Nutrition Examination Survey (NHANES) (2017–2018 cycle).

## Methods

### Study population

The NHANES is a representative US national population survey, which is non-institutionalized using a complicated, multilevel, probability sampling design (21). All trial subjects completed an informed consent form after the National Center for Health Statistics (NCHS) Research Ethics Review Board authorized the investigation strategy for NHANES.

### Study design

**Figure 1:**
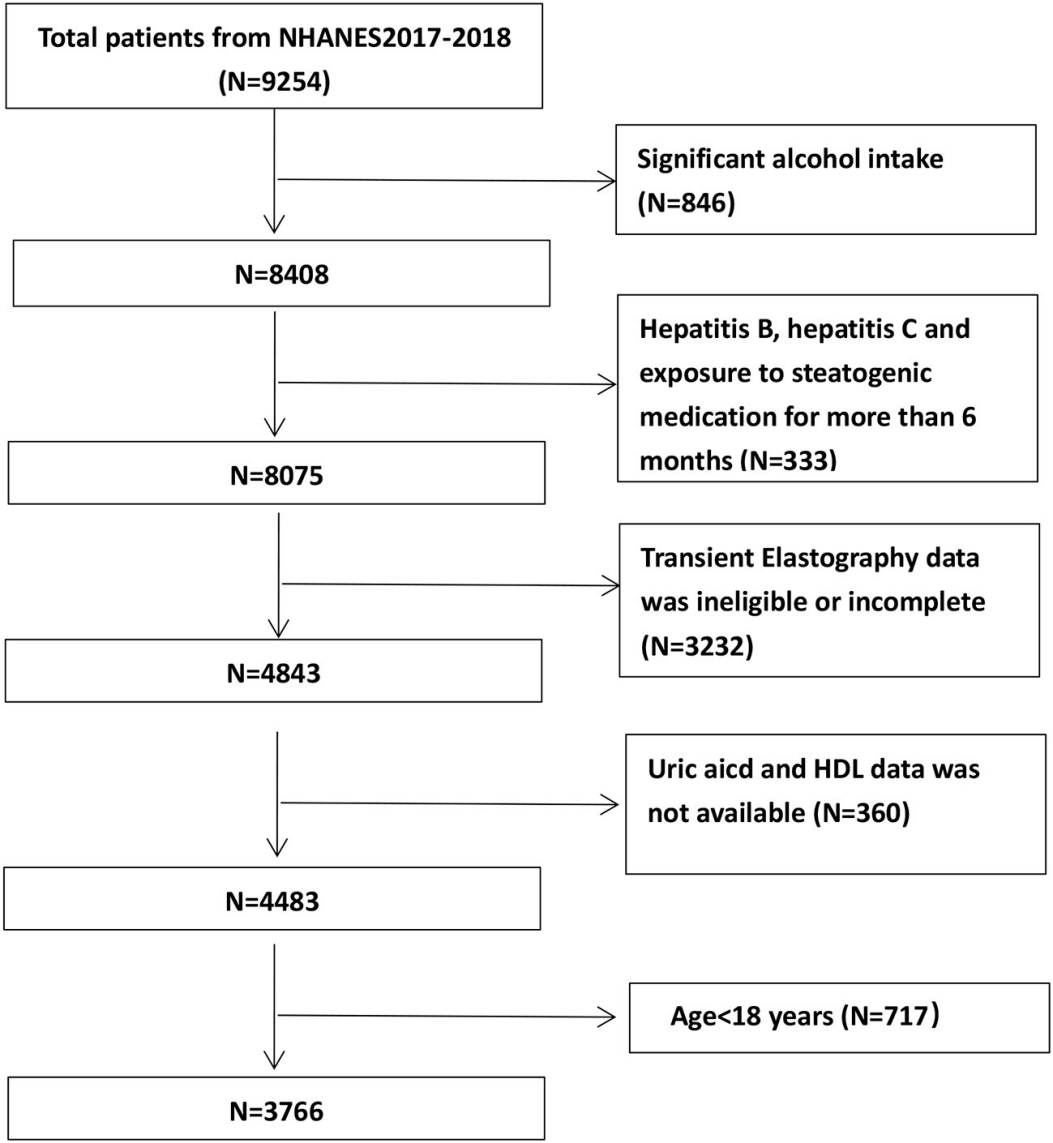
Flow-chart of the study samples.

Our investigation depended on information from the NHANES 2017–2018 cycle. We eliminated 846 individuals with excessive alcohol drinking (>14 drinks/week for female subjects and >21 drinks/week for male subjects), as well as 333 people with viral hepatitis B or C and those with steatogenic medication history for over 6 months (22). Then, we excluded the 3,592 individuals who had missing HDL-C, sUA, and transient elastography (TE) data. A final sample of 3,766 was obtained after 717 individuals under the age of 18 were excluded (Figure 1).

### Vibration controlled transient elastography (VCTE)

The most reliable way to diagnose NAFLD and assess the degree of hepatic steatosis and fibrosis is typically by liver biopsy. However, it is impractical to perform liver biopsies on large patient cohorts due to its acceptability, cost, and risk (23). In recent years, VCTE has been considered the best non-invasive and affordable tool, which was widely used for hepatic fibrosis and steatosis evaluation in chronic liver diseases (24–26). Current clinical evidence suggests that the controlled attenuation parameter (CAP) and liver stiffness measurement (LSM) values increase with liver steatosis and fibrosis severity, respectively (27, 28). VCTE outcome was considered valid when at least 10 LSMs were acquired with more than 3 h of fasting time and <30% interquartile (IQR) range/median (29).

### The exposure and outcome variables definition

The exposure variable was the UHR, which was determined as sUA divided by serum HDL-C. The values of CAP and LSM were two continuous outcome variables evaluated by VCTE, while the NAFLD status and the liver fibrosis stages were considered categorical outcome variables. CAP values ≥ 288 dB/m were considered to define NAFLD status (30). We defined liver fibrosis stages based on median LSM cutoff values: fibrotic non-alcoholic steatohepatitis (NASH) (F2) as 8.0 to <12.0 kPa, advanced fibrosis (F3) as 12.0 to <20.0 kPa, and cirrhosis (F4) as ≥20 kPa (30).

### Covariates

The pretense of systolic blood pressure (BP) values ≥ 140 mmHg and/or diastolic BP values ≥ 90 mmHg, self-reported hypertension, and/or the use of hypertensive medications were used to define hypertension (26). One of the following characteristics was used to diagnose T2DM (31): (1) self-reported diabetes; (2) glucose-lowering drugs or insulin usage; (3) 126 mg/dl (7 mmol/L) or more fasting plasma glucose (FPG) value; and (4) 6.5% or more hemoglobin A1c (HA1c) value. Smoking status was divided into three categories by self-reported questionnaire (non-smokers, former smokers, and current smokers). Depending on the metabolic equivalents (MET-minutes), physical activity was classified as low, moderate, high, or very high (32). Detailed information on other covariates can be obtained from http://www.cdc.gov/nchs/nhanes/.

### Statistical analysis

A weighted analysis was conducted as advised by the NCHS to achieve national representation. While continuous data were shown as weighted mean ± standard deviation (SD), categorical variables were provided as weighted proportions. The UHR was divided into five groups called quintiles. We used the weighted χ^2^ test for categorical variables and a weighted linear regression model for continuous variables to assess the difference between each group. Then, we used a weighted multivariate logistic regression model to investigate the relationship between UHR and NAFLD status as well as liver fibrosis stages. The connection between UHR and liver steatosis and fibrosis severity based on liver CAP and LSM values was investigated using weighted multivariate linear regression analysis. Furthermore, we performed a subgroup analysis on sex. Three models were constructed: model 1: no covariates were adjusted; model 2: age, gender, and race were adjusted; and model 3: age, gender, race, BMI, diabetes status, hypertension, smoking status, activity level, antihyperlipidemic medication, triglyceride (TG), gamma-glutamyl transpeptidase (GGT), aspartate aminotransferase (AST), alanine aminotransferase (ALT), alkaline phosphatase (ALP), total cholesterol (TC), serum albumin, total bilirubin, creatine, and glycosylated hemoglobin A1c (HbA1c) were adjusted. To identify any potential non-linear relationships between UHR and NAFLD probabilities, smooth curving fits and generalized additive models were utilized. Finally, ROC curve studies were employed to compare UHR to uric acid and HDL. Data analysis in our study was performed using EmpowerStats software (http://www.empowerstats.com) and R (http://www.R-project.org). Statistics were regarded as significant at a *P*-value of < 0.05.

## Results

### Characteristics of the study participants

Table 1 describes the weighted features of the 3,766 subjects based on UHR quintiles. Substantial differences were observed between UHR quintiles and baseline features. Individuals in the greater quintile groups were more likely to be men, smoke more, and have elevated rates of NAFLD, hypertension, and T2DM compared to the group of Q1. Meanwhile, they had increased waist circumference (WC), BMI, HbA1c, TG, AST, ALT, AKP, GGT, serum albumin, total bilirubin, serum creatinine, uric acid, CAP value, and LSM value. However, no significant racial or age variations were identified. Supplementary Table S1 illustrates the participants' baseline features depending on the status of NAFLD. In the NAFLD group, the UHR of the individuals was more than those in the non-NAFLD group (*P* < 0.0001).

**Table 1:** Weighted characteristics of five groups.

**Characteristics**	**Q1 (1.36-7.21) *N =* 753**	**Q2 (7.21-9.27) *N =* 751**	**Q3 (9.29-11.79) *N =* 756**	**Q4 (11.80-15.31) *N =* 752**	**Q5 (15.33-46.67) *N =* 754**	***P*-value**
NAFLD (%)						<0.0001
No	84.1	75.2	66.3	57.9	39	
Yes	15.9	24.8	33.7	42.1	61	
Age (years)	49.0 ± 17.3	48.4 ± 18.3	49.2 ± 18.0	49.4 ± 17.4	48.4 ± 17.8	0.7453
Gender (%)						<0.0001
Male	12.2	34.7	50.2	71.2	84.2	
Female	87.8	65.3	49.8	28.8	15.8	
RACE (%)						0.1254
Non-Hispanic White	65.3	58.6	64.2	62.7	61.5	
Non-Hispanic Black	12.1	13	11.3	10.9	9.8	
Hispanic	5.9	9.5	7.2	8.7	9	
Other Race	16.7	19	17.4	17.7	19.7	
Smoking behavior (%)						<0.0001
Current smoke	11.9	15.2	12.1	15.3	10.4	
Ever smoke	19.4	21.8	29.2	25.3	32	
Never smoke	68.6	63	58.7	59.4	57.6	
Hypertension (%)						<0.0001
No	70.2	64	59.9	59.8	48	
Yes	29.8	36	40.1	40.2	52	
T2DM						<0.0001
No	91.7	87.8	84.2	78.8	75.9	
Yes	8.3	12.2	15.8	21.2	24.1	
BMI (Kg/m^2^)	25.9 ± 5.8	28.6 ± 6.5	30.1 ± 7.2	30.8 ± 6.8	33.5 ± 7.3	<0.0001
WC (cm)	89.5 ± 14.0	96.8 ± 14.9	101.0 ± 15.8	104.8 ± 15.6	111.4 ± 16.2	<0.0001
ALT (IU/L)	17.8 ± 14.7	19.6 ± 14.7	21.4 ± 14.1	25.1 ± 15.0	28.9 ± 18.4	<0.0001
AST (IU/L)	20.2 ± 10.8	21.0 ± 12.5	21.4 ± 13.0	22.2 ± 9.1	23.7 ± 11.2	<0.0001
ALP (IU/L)	74.2 ± 25.3	77.6 ± 25.6	79.6 ± 24.1	78.7 ± 23.4	78.4 ± 24.0	<0.0001
GGT (IU/L)	22.3 ± 31.8	24.8 ± 30.7	26.7 ± 31.4	31.8 ± 33.4	35.7 ± 33.3	<0.0001
Albumin (mg/dL)	41.0 ± 2.9	40.5 ± 3.3	41.0 ± 3.3	41.3 ± 3.1	41.3 ± 3.2	<0.0001
Total bilirubin (mg/dL)	0.4 ± 0.2	0.4 ± 0.3	0.5 ± 0.3	0.5 ± 0.3	0.5 ± 0.3	<0.0001
Serum creatinine (mg/dL)	0.8 ± 0.2	0.8 ± 0.2	0.9 ± 0.3	1.0 ± 0.3	1.0 ± 0.5	<0.0001
HbA1c (%)	5.6 ± 0.9	5.7 ± 1.0	5.7 ± 1.0	5.8 ± 0.9	5.9 ± 1.1	<0.0001
Total cholesterol (mg/dL)	192.3 ± 38.2	188.4 ± 38.0	188.5 ± 40.7	185.7 ± 41.8	188.1 ± 42.5	0.0252
Triglyceride (mg/dL)	93.4 ± 42.6	114.6 ± 54.7	132.3 ± 71.8	156.9 ± 81.3	230.3 ± 187.5	<0.0001
HDL (mg/dL)	69.2 ± 13.0	57.9 ± 10.2	51.4 ± 8.1	44.8 ± 7.0	38.4 ± 6.5	<0.0001
Uric acid (mg/dL)	3.9 ± 0.8	4.8 ± 0.8	5.4 ± 0.8	5.9 ± 0.9	7.1 ± 1.2	<0.0001
UHR (%)	5.7 ± 1.1	8.3 ± 0.6	10.6 ± 0.7	13.3 ± 1.0	18.8 ± 3.7	<0.0001
LSM (kPa)	4.9 ± 3.0	5.4 ± 3.5	5.7 ± 4.6	6.2 ± 6.0	7.2 ± 6.6	<0.0001
CAP (dB/m)	232.2 ± 54.2	249.8 ± 58.0	264.6 ± 56.6	278.4 ± 59.0	301.2 ± 60.4	<0.0001

Mean ± SD was for continuous variables. The p-value was calculated by the weighted linear regression model. % was for categorical variables. The p-value was calculated by the weighted chi-square test. NAFLD, non-alcoholic fatty liver disease; T2DM, type 2 diabetes mellitus; BMI, body mass index; WC, waist circumference; ALT, alanine aminotransferase; AST, aspartate aminotransferase; ALP, alkaline phosphatase; GGT, gamma-glutamyl transpeptidase; HbA1c, glycosylated hemoglobin A1c; HDL, high-density lipoprotein; UHR, serum uric acid-to-high-density lipoprotein cholesterol ratio; LSM, liver stiffness measurement; CAP, controlled attenuation parameter.

### Association between UHR and NAFLD

**Figure 2:**
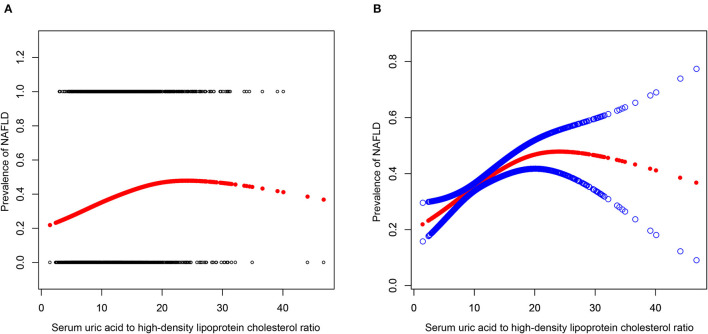
Associations between serum uric acid to high-density lipoprotein cholesterol ratioand prevalence of NAFLD. **(A)** Each black point represents a sample. **(B)** Solid redline represents the smooth curve fit between variables. Blue bands represent the 95% of confidence interval from the fit. Adjusted for: age, gender, race, hypertension, BMI, dyslipidemia drug, T2DM, smoke, physical activity, ALT, AST, AKP, GGT, TC, TG, Serum creatinine, albumin, Total bilirubin, and HbA1c. NAFLD, Non-alcoholic fatty liver disease; UHR, serum uric acid-to-high-density lipoprotein cholesterol ratio; BMI, Body mass index; ALT, Alanine aminotransferase; AST, Aspartate aminotransferase; ALP, Alkaline phosphatase; GGT, Gamma-glutamyl transpeptidase; TC, Total cholesterol; TG, Triglyceride; HbA1c, Glycosylated hemoglobin A1c; HbA1c, glycosylated hemoglobin A1c.

A multivariate regression analysis was performed between NAFLD prevalence and UHR (Table 2). UHR was statistically positively related to the NAFLD probabilities in all three models: model 1 (OR = 2.139, 95% CI: 1.870, 2.447), model 2 (OR = 2.513, 95% CI: 2.137, 2.956), and model 3 (OR = 1.331, 95% CI: 1.100, 1.611). Additionally, subjects in quintiles 2, 3, 4, and 5 presented a 28.5, 54.9, 59.4, and 122.9% increase in NAFLD risks, respectively, compared with the lowest level of UHR (Q1) in model 3 (P for trend <0.001). This result indicated that people with raised UHR were more prone to develop NAFLD than those with reduced UHR. After adjustment for all variables in subgroup analyses stratified by gender, the positive connection of UHR and NAFLD risk persisted in female subjects (OR = 1.627, 95% CI: 1.238, 2.139), but not in male subjects (OR = 1.201, 95% CI: 0.943, 1.529). We further characterized the non-linear relationship between UHR and NAFLD status utilizing a generalized additive model and smooth curve fittings (Figure 2). The result demonstrated that a UHR between 20 and 30% suggested a saturation NAFLD risk impact, and a value below 20% might indicate a linear association with the NAFLD risk.

**Table 2:** Associations between UHR and NAFLD status in logistic regression analysis.

	**Model 1 OR (95% CI), *P*-value**	**Model 2 OR (95% CI), *P*-value**	**Model 3 OR (95% CI), *P*-value**
UHR (per SD increase)	2.139 (1.870, 2.447) <0.001	2.513 (2.137, 2.956) <0.001	1.331 (1.100, 1.611) 0.003
Q1 (1.36–7.21)	Reference	Reference	Reference
Q2 (7.21–9.27)	1.934 (1.500, 2.493) <0.001	1.989 (1.535, 2.578) <0.001	1.285 (0.959, 1.722) 0.093
Q3 (9.29–11.79)	2.943 (2.301, 3.764) <0.001	3.085 (2.388, 3.984) <0.001	1.549 (1.156, 2.076) 0.003
Q4 (11.80–15.31)	3.990 (3.128, 5.089) <0.001	4.306 (3.319, 5.587) <0.001	1.594 (1.174, 2.164) 0.003
Q5 (15.33–46.67)	7.974 (6.247, 10.178) <0.001	9.286 (7.087, 12.168) <0.001	2.229 (1.589, 3.126) <0.001
P for trend	<0.001	<0.001	<0.001
**Subgroup analysis stratified by sex**			
Men	1.969 (1.642, 2.362) <0.001	2.052 (1.701, 2.478) <0.001	1.201 (0.943, 1.529) 0.139
Women	3.190 (2.556, 3.983) <0.001	3.595 (2.825, 4.575) <0.001	1.627 (1.238, 2.139) <0.001

Model 1: no covariates were adjusted. Model 2: age, gender, and race were adjusted. Model 3: age, gender, race, hypertension, BMI, dyslipidemia drug, T2DM, smoke, physical activity, ALT, AST, AKP, GGT, TC, TG, serum creatinine, albumin, total bilirubin, and HbA1c were adjusted. NAFLD, non-alcoholic fatty liver disease; UHR serum uric acid-to-high-density lipoprotein cholesterol ratio; BMI, body mass index; T2DM, type 2 diabetes mellitus; ALT, alanine aminotransferase; AST, aspartate aminotransferase; ALP, alkaline phosphatase; GGT, gamma-glutamyl transpeptidase; TC, total cholesterol; TG, triglyceride; HbA1c, glycosylated hemoglobin A1c; HbA1c, glycosylated hemoglobin A1c.

### Association between UHR and the severity of liver steatosis and fibrosis

Table 3 demonstrates the relationships between UHR and hepatic steatosis according to CAP values. UHR was evidenced to be dramatically and positively connected to the severity of hepatic steatosis in model 1 (β = 24.456, 95% CI: 22.542, 26.370), model 2 (β = 25.654, 95% CI: 23.535, 27.774), and model 3 (β = 6.070, 95% CI: 3.896, 8.244), with a P for trend of <0.001 (Table 3).

**Table 3:** Associations between UHR and CAP value in linear regression analysis.

	**Model 1 β (95% CI), *P*-value**	**Model 2 β (95% CI), *P*-value**	**Model 3 β (95% CI), *P*-value**
UHR (per SD increase)	24.456 (22.542, 26.370) <0.001	25.654 (23.535, 27.774) <0.001	6.070 (3.896, 8.244) <0.001
Q1 (1.36–7.21)	Reference	Reference	Reference
Q2 (7.21–9.27)	17.635 (11.864, 23.405) <0.001	19.243 (13.583, 24.903) <0.001	3.143 (-1.624, 7.911) 0.196
Q3 (9.29–11.79)	32.422 (26.823, 38.021) <0.001	34.534 (28.898, 40.170) <0.001	8.002 (3.122, 12.882) 0.001
Q4 (11.80–15.31)	46.166 (40.470, 51.862) <0.001	49.177 (43.143, 55.211) <0.001	12.625 (7.254, 17.995) <0.001
Q5 (15.33–46.67)	69.023 (63.247, 74.799) <0.001	73.581 (67.239, 79.924) <0.001	18.295 (12.073, 24.518) <0.001
P for trend	<0.001	<0.001	<0.001

Model 1: no covariates were adjusted. Model 2: age, gender, and race were adjusted. Model 3: age, gender, race, hypertension, BMI, dyslipidemia drug, T2DM, smoke, physical activity, ALT, AST, AKP, GGT, TC, TG, serum creatinine, albumin, total bilirubin, and HbA1c were adjusted. NAFLD, non-alcoholic fatty liver disease; UHR, serum uric acid-to-high-density lipoprotein cholesterol ratio; BMI, body mass index; T2DM, type 2 diabetes mellitus; ALT, alanine aminotransferase; AST, aspartate aminotransferase; ALP, alkaline phosphatase; GGT, gamma-glutamyl transpeptidase; TC, total cholesterol; TG, triglyceride; HbA1c, glycosylated hemoglobin A1c; HbA1c, glycosylated hemoglobin A1c.

We further examined the association between UHR and liver fibrosis stages. Our analysis revealed that UHR was significantly associated with fibrotic NASH, significant fibrosis, and cirrhosis in both model 1 and model 2, but not in model 3 (fibrotic NASH: OR =1.003, 95% CI: 0.825, 1.220; significant fibrosis: OR = 1.306, 95% CI: 0.991, 1.720; cirrhosis: OR = 1.032, 95% CI: 0.693, 1.537) (Table 4). Similarly, we observed a positive relationship between UHR and hepatic fibrosis severity based on LSM values in model 1 (β = 0.841, 95% CI: 0.679, 1.004) and model 2 (β = 0.885, 95% CI: 0.698, 1.071). However, after adjusting for all potential confounders, this association was no longer significant (β = 0.034, 95% CI: 0.183, 0.251), with a P for trend of 0.828 (Supplementary Table S2).

**Table 4:** Associations between UHR and fibrosis stages in logistic regression analysis.

	**Model 1 OR (95% CI), *P*-value**	**Model 2 OR (95% CI), *P*-value**	**Model 3 OR (95% CI), *P*-value**
Fibrotic NASH (≥F2) LSM ≥ 8.0 kPa	1.557 (1.352, 1.793) <0.001	1.628 (1.387, 1.911) <0.001	1.003 (0.825, 1.220) 0.974
Advanced fibrosis (≥F3) LSM ≥ 12.0 kPa	1.669 (1.400, 1.990) <0.001	1.782 (1.497, 2.123) <0.001	1.306 (0.991, 1.720) 0.058
Cirrhosis (≥F4) LSM ≥ 20.0 kPa	2.139 (1.869, 2.447) <0.001	2.513 (2.137. 2.956) <0.001	1.032 (0.693, 1.537) 0.878

Model 1: no covariates were adjusted. Model 2: age, gender, and race were adjusted. Model 3: age, gender, race, hypertension, BMI, dyslipidemia drug, T2DM, smoke, physical activity, ALT, AST, AKP, GGT, TC, TG, serum creatinine, albumin, total bilirubin, and HbA1c were adjusted. NAFLD, non-alcoholic fatty liver disease; UHR, serum uric acid-to-high-density lipoprotein cholesterol ratio; BMI, body mass index; T2DM, type 2 diabetes mellitus; ALT, alanine aminotransferase; AST, aspartate aminotransferase; ALP, alkaline phosphatase; GGT, gamma-glutamyl transpeptidase; TC, total cholesterol; TG, triglyceride; HbA1c, glycosylated hemoglobin A1c; HbA1c, glycosylated hemoglobin A1c.

### ROC analysis

**Figure 3:**
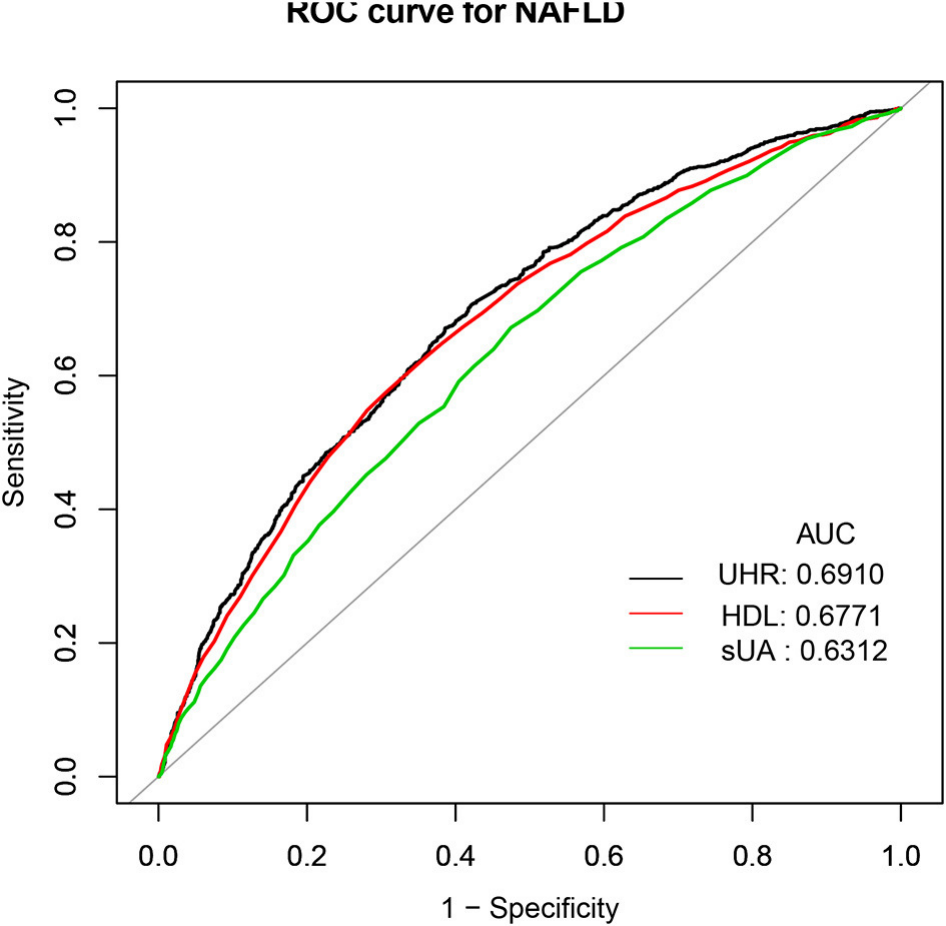
ROC curves for UHR, compared to sUA and HDL alone. ROC, Receiver operating characteristic; UHR, serum uric acid-to-high-density lipoprotein.

The receiver operating characteristic curve of the UHR, sUA, and HDL capability to anticipate NAFLD risk is illustrated in Figure 3. Supplementary Table S3 shows that the area under the curve (AUC) for UHR in the ROC analysis was 0.6910 (95% CI: 0.6737–0.7083), which was considerably higher than that of sUA and HDL (*P* < 0.0001). This suggested that UHR may be a more suitable indicator for NAFLD compared to sUA or HDL alone, although the diagnostic accuracy is still limited.

## Discussion

An elevated UHR value was shown to be substantially correlated with NAFLD risk in this cross-sectional study, which examined the association between NAFLD prevalence and UHR in a large, national American adult population. After fully adjusting for all possible confounders, we identified a 1.331-fold increase in NAFLD risk per each SD increase in UHR. In the subgroup analysis, it is noteworthy that statistically significant relationships persisted exclusively in female subjects rather than in male subjects. We further revealed a positive correlation between hepatic steatosis degree and UHR. After accounting for all the probable covariates, however, the association between liver fibrosis degree and UHR was no longer significant. Moreover, the ROC analysis outcomes exhibited that UHR was more effective in detecting NAFLD than either sUA or HDL alone. These results indicated that UHR could be a new and applicable marker for identifying individuals at higher risk for NAFLD. To the best of our knowledge, this investigation has the largest sample size available on the association between UHR and NAFLD status in the American population.

The association between NAFLD status and sUA has been widely recognized in the literature. In 2002, authors initially discovered a considerable relationship between NAFLD prevalence and sUA (33). Since then, some studies confirmed that raised sUA levels were related to an elevated NAFLD risk (34, 35). Simultaneously, researchers have found that low HDL-C was a main lipid disturbance, which was strongly connected to the NAFLD severity and progression as well (12, 13). However, more recently, the HDL cholesterol and uric acid combination have been suggested as a novel and more sensitive biomarker of metabolic and inflammatory disorders (36). Kocak et al. demonstrated that the UHR was superior to all other recognized criteria including uric acid as a marker of MetS (36). Given the fact that hepatic steatosis and metabolic syndrome are closely related (37), the UHR is expected to increase in NAFLD subjects as well. Currently, we found three limited pieces of literature about UHR and NAFLD status. One study included 6,285 lean Chinese adults who participated in their yearly health examinations through the year 2019 (20). The study found that the UHR was independently related to an elevated NAFLD risk (odds ratio: 1.105; 95% CI: 1.076–1.134; *P* < 0.001) after adjusting for potential confounders. However, this association was only applied to lean adults. At Wenzhou People's Hospital, 9,837 non-obese Chinese individuals with normal lipid profiles participated in a 5-year retrospective cohort study (18). The multivariate Cox proportional hazard regression analyses revealed that participants with elevated UHR values (Q5) had a much higher risk for the incidence of NAFLD than those with reduced UHR (Q1–4). They reported that the AUC of the UHR was greater than that of HDL-C and UA. Additionally, Kosekli et al. proposed that elevated UHR could be considered an indicator of NAFLD occurrence in their small sample cross-sectional study involving 117 subjects in Turkey (19). Our results were consistent with the above studies. We verified the positive association between NAFLD and UHR in a large population-based study of the general American adult. Additionally, we performed an ROC analysis and observed that the diagnostic accuracy of UHR for NAFLD was low, despite its improved performance compared to sUA and HDL-C alone. Therefore, elevated UHR can be considered an independent risk factor for NAFLD development and may serve as a useful marker for assessing the risk of NAFLD occurrence. However, its diagnostic value for NAFLD is limited. Further studies are needed to validate these findings and explore the clinical implications of UHR in the diagnosis and management of NAFLD.

Notably, we used CAP values evaluated by VCTE to define NAFLD, rather than ultrasound used in the above studies. Although conventional liver ultrasound is commonly used in clinical practice, it has limited sensitivity in detecting mild steatosis, especially in patients with obesity (38, 39), and provides only a subjective semiquantitative assessment of steatosis severity. The absence of detectable steatosis on ultrasound does not necessarily exclude the presence of NASH or fibrosis. As a result, it is not recommended as a reliable tool for identifying hepatic steatosis due to its low sensitivity for NAFLD (30). VCTE has emerged as a significant tool for evaluating patients with NAFLD (40). The accuracy of the CAP value measured by VCTE in detecting hepatic steatosis has been validated against liver biopsy, although its accuracy in differentiating steatosis ≥33% and ≥66% is suboptimal (25, 40). Moreover, previous studies have demonstrated that CAP outperforms ultrasonography in detecting and grading liver steatosis (41, 42). Therefore, as the most widely available and well-evaluated point-of-care technique, transient elastography can be employed for identifying hepatic steatosis (30).

In comparison to the above studies, we further explored the subgroup analysis on sex. Interestingly, our data suggested that UHR related to NAFLD risk in female subjects but not in male subjects. Similar gender differences had been found in other related studies involving sUA and NAFLD. Wu et al. evaluated the correlation between sex-specific sUA levels and NAFLD in a large-scale study for Chinese adults by conducting a cross-sectional study (43). They demonstrated that the association between NAFLD and sUA was considerably more significant in female subjects than in male subjects. Another cross-sectional study identically revealed that the positive relationship between NAFLD prevalence and sUA/Cr level existed in female subjects rather than only in male subjects (44). Explaining the underlying mechanism of sex differences in our study, the result remains challenging. One potential partial explanation for this result could be hormonal differences, as hormones play a crucial role in various physiological processes, including metabolism and inflammation, which are closely associated with NAFLD development (43, 45). Additionally, genetic factors and sex-specific differences in gene expression may also contribute to the observed gender differences (46, 47). It is possible that there are sex-specific genetic differences that interact with hormonal differences, leading to differential associations between UHR and NAFLD risk in male and female subjects. Further investigations need to be performed. In addition, we intriguingly discovered a non-linear correlation between UHR and NAFLD that has never been reported to date. There was probably a saturating effect of NAFLD risk with a UHR of 20% to 30%. Our findings may suggest new ideas for the prevention and treatment of NAFLD.

Importantly, no previous studies reported the association between UHR level and hepatic steatosis and fibrosis severity. Prior investigations have reported controversial outcomes concerning the relationship between liver steatosis and fibrosis and sUA (48–50). The research conducted by Lee illustrated a positive relationship between sUA levels and hepatic steatosis in Korean adults (48). Meanwhile, Duan et al. have found that sUA was positively correlated with CAP and LSM values, suggesting the positive connection between sUA and hepatic steatosis and fibrosis degree (49). Conversely, Takashi Nakahara et al. revealed an inverse correlation between sUA levels and fibrosis stages in Japanese adults (50). These opposite results may be related to different patient populations. In our research, we detected a significant relation between UHR level and hepatic steatosis severity based on CAP value, suggesting that the UHR level might be a promising biomarker for liver steatosis management in patients with NAFLD. However, the UHR level and hepatic fibrosis were not independently associated. This difference in the findings could be attributed to the fact that our exposure variable included both sUA and high-density lipoprotein, which differs from the exposure variable used in the previously mentioned studies. Additionally, we adjusted for several important covariates, including physical activity and various biochemical indicators, to improve the precision of our analysis. However, LSM is not the gold standard to detect fibrosis in NAFLD. Further investigations are necessary.

The potential pathway of the relationship between NAFLD risk and sUA was related to inflammation, NADPH oxidase subunit-4 (NOX4) associated lipogenesis, production of reactive oxygen species (ROS), NOD-like receptor family pyrin domain containing 3 (NLRP3)-related inflammasome activation, and a cascade of endoplasmic reticulum stress via sterol regulatory element-binding protein 1 (SREBP-1) (11). Meanwhile, HDL-C could prevent monocyte migration to inhibit pro-inflammatory cytokines expression and adhesion molecules (51). It showed both antioxidant and anti-inflammatory properties. Therefore, the combination of sUA and HDL-C (UHR) could raise the burden of inflammation and oxidative stress, and further predict NAFLD by reflecting insulin sensitivity (16, 52).

The advantage of our investigation includes the big sample size and the national representativeness of the US. We also considered many potential confounding factors, such as age, gender, BMI, T2DM, hypertension, smoking status, and physical activity. However, there are several limitations associated with our study. First, owing to the cross-sectional nature of this investigation, it was limited to define causality. Second, NAFLD and liver fibrosis stages were not defined using the gold standard liver biopsy, but rather by CAP and LSM values. Third, some self-reported confounders are vulnerable to biased recall. Additionally, we may miss some possibly confounding variables; there is still a chance of bias.

## Conclusion

In conclusion, in a large American population, we suggested that the elevated UHR level was independently related to an increased NAFLD risk and liver steatosis severity. These results highlight the potential of UHR as a relevant biomarker for identifying individuals at higher risk for NAFLD and may contribute to early detection and intervention strategies for this prevalent liver condition.

## Data availability statement

The datasets presented in this study can be found in online repositories. The names of the repository/repositories and accession number(s) can be found at: https://www.cdc.gov/nchs/nhanes.

## Ethics statement

The studies involving human participants were reviewed and approved by the National Center for Health Statistics (NCHS) Research Ethics Review Board approved the investigation protocol for NHANES and all study participants signed an informed consent term. The informed consent procedures for all participants are publicly available through the CDC.gov website. The patients/participants provided their written informed consent to participate in this study. Written informed consent was obtained from the individual(s) for the publication of any potentially identifiable images or data included in this article.

## Author contributions

YX and WK conceived and designed the study. YW, ZW, and YX collected the data. XZ, KH, and WK completed the statistical analyzes and analyzed the data. YX, XZ, and KH drafted and revised the manuscript. GQ and JC critically reviewed the manuscript. All authors have given the final approval of the manuscript.
