# Supplementary material for: Association of serum uric acid-to-high-density lipoprotein cholesterol ratio with non-alcoholic fatty liver disease in American adults: a population-based analysis
Source: Front Med (Lausanne). 2023 May 15;10:1164096. doi: 10.3389/fmed.2023.1164096 (PMC10225665; doi:10.3389/fmed.2023.1164096)
Supplement: Supplementary file 1 [file Table_1.docx]

Supplemental Table1 Weighted characteristics of the NAFLD and non-NAFLD groups

| **Characteristics** | **Non-NAFLD** | **NAFLD** | **P value** |
| --- | --- | --- | --- |
| Age(years) | 46.8 ± 18.3 | 52.9 ± 15.9 | <0.0001 |
| Gender (%) |  |  | <0.0001 |
| Male | 45.8 | 55.7 |  |
| Female | 54.2 | 44.3 |  |
| RACE (%) |  |  | <0.0001 |
| Non-Hispanic White | 61.3 | 65 |  |
| Non-Hispanic Black | 12.8 | 8.8 |  |
| Hispanic | 6.5 | 10.6 |  |
| Other Race | 19.4 | 15.5 |  |
| Smoking behavior (%) |  |  | 0.0004 |
| Current smoke | 13.6 | 11.8 |  |
| Ever smoke | 23.4 | 29.2 |  |
| Never smoke | 63.1 | 59 |  |
| Hypertension (%) |  |  | <0.0001 |
| No | 69.8 | 43.8 |  |
| Yes | 30.2 | 56.2 |  |
| T2DM |  |  | <0.0001 |
| No | 91.8 | 69.1 |  |
| Yes | 8.2 | 30.9 |  |
| BMI (Kg/m^2^) | 27.1 ± 5.7 | 34.3 ± 7.2 | <0.0001 |
| WC (cm) | 93.9 ± 14.0 | 112.5 ± 15.3 | <0.0001 |
| ALT (IU/L) | 19.8 ± 14.3 | 27.3 ± 17.5 | <0.0001 |
| AST (IU/L) | 20.9 ± 10.5 | 23.1 ± 13.0 | <0.0001 |
| ALP(IU/L) | 75.6 ± 24.3 | 81.4 ± 24.6 | <0.0001 |
| GGT (IU/L) | 23.5 ± 24.5 | 36.5 ± 42.4 | <0.0001 |
| Albumin(mg/dL) | 41.3 ± 3.1 | 40.6 ± 3.1 | <0.0001 |
| Total bilirubin(mg/dL) | 0.5 ± 0.3 | 0.4 ± 0.3 | <0.0001 |
| Serum creatinine (mg/dL) | 0.9 ± 0.3 | 0.9 ± 0.4 | 0.085 |
| HbA1c (%) | 5.5 ± 0.7 | 6.1 ± 1.2 | <0.0001 |
| Total cholesterol (mg/dL) | 187.6 ± 39.9 | 190.9 ± 40.9 | 0.0156 |
| Triglyceride (mg/dL) | 120.7 ± 87.3 | 185.8 ± 132.5 | <0.0001 |
| HDL (mg/dL) | 55.9 ± 14.3 | 47.4 ± 12.5 | <0.0001 |
| Uric acid (mg/dL) | 5.1 ± 1.3 | 5.8 ± 1.4 | <0.0001 |
| UHR (%) | 10.0 ± 4.3 | 13.2 ± 5.0 | <0.0001 |
| LSM (kPa) | 5.0 ± 3.5 | 7.3 ± 6.7 | <0.0001 |
| CAP (dB/m) | 227.7 ± 39.4 | 332.4 ± 33.0 | <0.0001 |

Mean ± SD was for continuous variables. The p-Value was calculated by weighted linear regression model. % was for categorical variables. The p-Value was calculated by the weighted chi-square test. Abbreviations: NAFLD Non-alcoholic fatty liver disease, T2DM type 2 diabetes, BMI Body mass index, WC Waist circumference, ALT Alanine aminotransferase, AST Aspartate aminotransferase, ALP Alkaline phosphatase, GGT Gamma-glutamyl transpeptidase, HbA1c Glycosylated hemoglobin A1c, HDL High-density lipoprotein, UHR Serum Uric Acid to high-density lipoprotein cholesterol ratio, LSM Liver stiffness measurement, CAP Controlled attenuation parameter

Supplemental Table 2 Associations between UHR and liver stiffness in linear regression analysis

|  | **Model 1 *β* (95% CI), *P* value** | **Model 2 *β* (95% CI), *P* value** | **Model 3 *β* (95% CI), *P* value** |
| --- | --- | --- | --- |
| UHR (per SD increase) | 0.841 (0.679, 1.004) <0.001 | 0.885 (0.698, 1.071) <0.001 | 0.034 (-0.183, 0.251) 0.762 |
| Q1(1.36-7.21) | Reference | Reference | Reference |
| Q2(7.21-9.27) | 0.484 (-0.007, 0.975) 0.054 | 0.523 (0.025, 1.020) 0.040 | -0.394 (-0.870, 0.083) 0.105 |
| Q3(9.29-11.79) | 0.833 (0.357, 1.310) <0.001 | 0.885 (0.389, 1.380) <0.001 | -0.494 (-0.982, -0.007) 0.047 |
| Q4(11.80-15.31) | 1.279 (0.794, 1.764) <0.001 | 1.355 (0.825, 1.886) <0.001 | -0.383 (-0.920, 0.153) 0.161 |
| Q5(15.33-46.67) | 2.314 (1.822, 2.806) <0.001 | 2.430 (1.872, 2.988) <0.001 | -0.047 (-0.668, 0.574) 0.882 |
| P for trend | <0.001 | <0.001 | 0.828 |

Model 1: no covariates were adjusted. Model 2: age, gender, and race were adjusted. Model 3: age, gender, race, hypertension, BMI, dyslipidemia drug, T2DM, smoke, physical activity, ALT, AST, AKP, GGT, TC, TG, Serum creatinine, albumin, Total bilirubin, and HbA1c were adjusted. Abbreviations: NAFLD Non-alcoholic fatty liver disease, UHR Serum Uric Acid to high-density lipoprotein cholesterol ratio, BMI Body mass index, T2DM type 2 diabetes, ALT Alanine aminotransferase, AST Aspartate aminotransferase, ALP Alkaline phosphatase, GGT Gamma-glutamyl transpeptidase, TC Total cholesterol, TG Triglyceride, HbA1c Glycosylated hemoglobin A1c, HbA1c glycosylated hemoglobin A1c.

Supplemental Table 3 Areas under the receiver operating characteristic curves for each parameter in identifying nonalcoholic fatty liver disease

|  | AUC | 95% confidence interval | Best threshold | Specificity | Sensitivity |
| --- | --- | --- | --- | --- | --- |
| UHR | 0.6910 | 0.6737-0.7083 | 0.1063 | 0.6140 | 0.6711 |
| sUA | 0.6771* | 0.6593-0.6948 | 47.5000 | 0.6951 | 0.5734 |
| HDL | 0.6312* | 0.6129-0.6495 | 5.1500 | 0.5254 | 0.6718 |

Abbreviations: AUC area under the curve, UHR Serum Uric Acid to high-density lipoprotein cholesterol ratio, sUA serum uric acid, HDL High-density lipoprotein. *P < 0.0001, compare with UHR.
